# 3-Allyl-1,5-dibenzyl-1,5-benzodiazepine-2,4-dione

**DOI:** 10.1107/S160053680904851X

**Published:** 2009-11-21

**Authors:** Hind Jabli, Y. Kandri Rodi, Natalie Saffon, El Mokhtar Essassi, Seik Weng Ng

**Affiliations:** a Laboratoire de Chimie Organique Appliquée, Faculté des Sciences et Techniques, Université Sidi Mohamed Ben Abdallah, Fés, Morocco; b Service Commun Rayons-X FR2599, Université Paul Sabatier, Bâtiment 2R1, 118 route de Narbonne, Toulouse, France; c Laboratoire de Chimie Organique Hétérocyclique, Pôle de compétences Pharmacochimie, Université Mohammed V-Agdal, B.P. 1014 Avenue Ibn Batout, Rabat, Morocco; d Department of Chemistry, University of Malaya, 50603 Kuala Lumpur, Malaysia

## Abstract

The title compound, C_26_H_24_N_2_O_2_, features a benzene ring fused with a seven-membered diazepine ring; the latter ring adopts a boat conformation (with the allyl­dimethyl­amino­methyl-bearing C atom as the prow and the fused-ring C atoms as the stern).

## Related literature

For the crystal structure of benzo­diazepin-2,4-dione, see: Négrier *et al.* (2006 ▶).
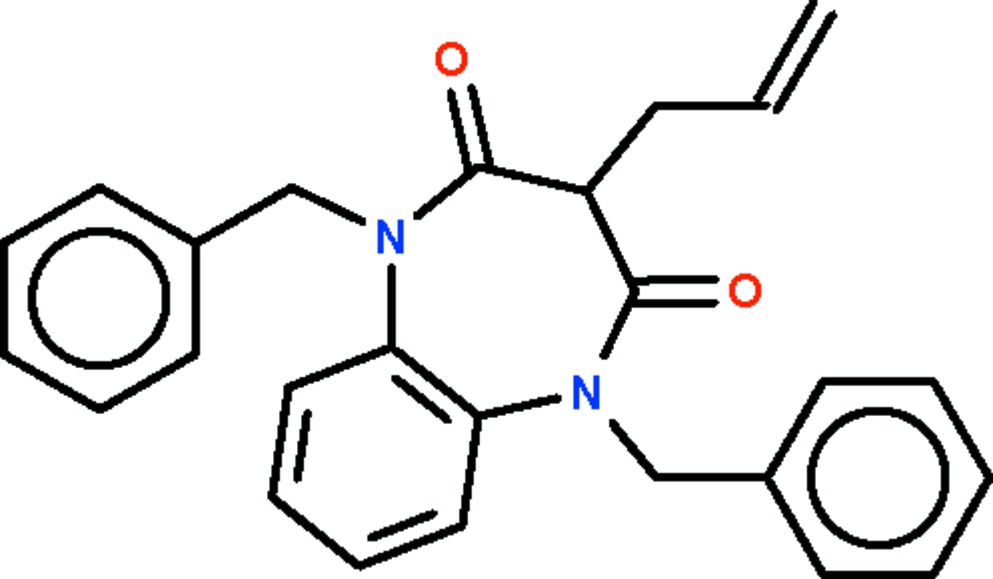


## Experimental

### 

#### Crystal data

C_26_H_24_N_2_O_2_
*M* *_r_* = 396.47
Monoclinic, 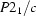

*a* = 9.2603 (4) Å
*b* = 14.0037 (6) Å
*c* = 16.2249 (7) Å
β = 91.996 (1)°
*V* = 2102.7 (2) Å^3^
*Z* = 4
Mo *K*α radiation
μ = 0.08 mm^−1^
*T* = 193 K
0.40 × 0.40 × 0.07 mm

#### Data collection

Bruker APEXII diffractometer
Absorption correction: none
27620 measured reflections
4820 independent reflections
2934 reflections with *I* > 2σ(*I*)
*R*_int_ = 0.050

#### Refinement

*R*[*F* ^2^ > 2σ(*F* ^2^)] = 0.043
*wR*(*F* ^2^) = 0.117
*S* = 1.00
4820 reflections
271 parameters
H-atom parameters constrained
Δρ_max_ = 0.21 e Å^−3^
Δρ_min_ = −0.18 e Å^−3^

### 

Data collection: *APEX2* (Bruker, 2005 ▶); cell refinement: *SAINT* (Bruker, 2005 ▶); data reduction: *SAINT*; program(s) used to solve structure: *SHELXS97* (Sheldrick, 2008 ▶); program(s) used to refine structure: *SHELXL97* (Sheldrick, 2008 ▶); molecular graphics: *X-SEED* (Barbour, 2001 ▶); software used to prepare material for publication: *publCIF* (Westrip, 2009 ▶).

## Supplementary Material

Crystal structure: contains datablocks global, I. DOI: 10.1107/S160053680904851X/sj2680sup1.cif

Structure factors: contains datablocks I. DOI: 10.1107/S160053680904851X/sj2680Isup2.hkl

Additional supplementary materials:  crystallographic information; 3D view; checkCIF report

## Figures and Tables

**Fig. 1.**
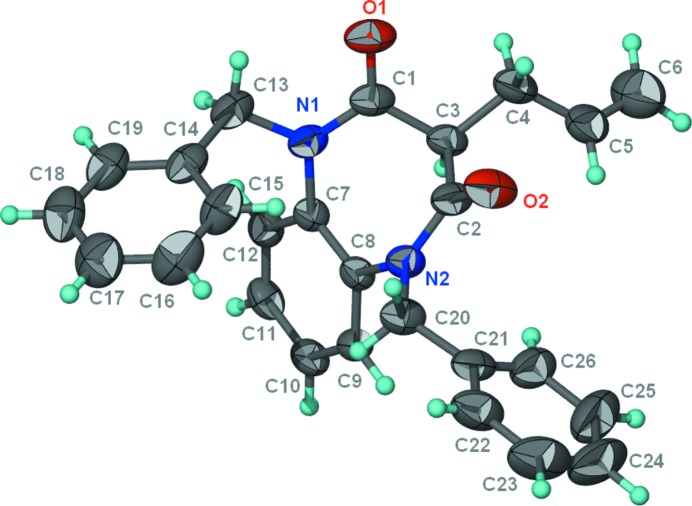
Thermal ellipsoid plot (Barbour, 2001) of C26H24N2O2 at the 70% probability level; hydrogen atoms are drawn as spheres of arbitrary radius.

## supplementary crystallographic information

### Experimental

To a solution of the potassium *t*-butoxide (0.24 g, 2.13 mmol) in THF (15 ml) was added 1,5-dibenzyl-1,5-benzodiazepine-2,4-dione (0.50 g, 1.40 mmol) and allyl bromide (0.20 ml, 1.88 mmol). Stirring was continued for 24 h. The reaction was monitored by thin layer chromatography. The mixture was filtered and the solution evaporated to give colorless crystals.

### Refinement

Carbon-bound H-atoms were placed in calculated positions (C—H 0.95 to 1.00 Å) and were included in the refinement in the riding model approximation, with *U*(H) set to 1.2*U*(C).

### Crystal data

**Table:**

C_26_H_24_N_2_O_2_	*F*(000) = 840
*M**_r_* = 396.47	*D*_x_ = 1.252 Mg m^−^^3^
Monoclinic, *P*2_1_/*c*	Mo *K*α radiation, λ = 0.71073 Å
Hall symbol: -P 2ybc	Cell parameters from 4269 reflections
*a* = 9.2603 (4) Å	θ = 2.5–25.0°
*b* = 14.0037 (6) Å	µ = 0.08 mm^−^^1^
*c* = 16.2249 (7) Å	*T* = 193 K
β = 91.996 (1)°	Plate, colorless
*V* = 2102.7 (2) Å^3^	0.40 × 0.40 × 0.07 mm
*Z* = 4	

### Data collection

**Table:**

Bruker APEXII diffractometer	2934 reflections with *I* > 2σ(*I*)
Radiation source: fine-focus sealed tube	*R*_int_ = 0.050
graphite	θ_max_ = 27.5°, θ_min_ = 1.9°
φ and ω scans	*h* = −11→12
27620 measured reflections	*k* = −18→18
4820 independent reflections	*l* = −21→16

### Refinement

**Table:**

Refinement on *F*^2^	Primary atom site location: structure-invariant direct methods
Least-squares matrix: full	Secondary atom site location: difference Fourier map
*R*[*F*^2^ > 2σ(*F*^2^)] = 0.043	Hydrogen site location: inferred from neighbouring sites
*wR*(*F*^2^) = 0.117	H-atom parameters constrained
*S* = 1.00	*w* = 1/[σ^2^(*F*_o_^2^) + (0.0487*P*)^2^ + 0.414*P*] where *P* = (*F*_o_^2^ + 2*F*_c_^2^)/3
4820 reflections	(Δ/σ)_max_ = 0.001
271 parameters	Δρ_max_ = 0.21 e Å^−^^3^
0 restraints	Δρ_min_ = −0.17 e Å^−^^3^

### Fractional atomic coordinates and isotropic or equivalent isotropic displacement parameters (Å^2^)

**Table:**

	*x*	*y*	*z*	*U*_iso_*/*U*_eq_	
O1	0.49018 (14)	0.26136 (9)	0.33899 (7)	0.0470 (3)	
O2	0.33507 (16)	0.40157 (8)	0.16934 (7)	0.0498 (4)	
N1	0.50477 (15)	0.15481 (9)	0.23332 (8)	0.0330 (3)	
N2	0.37563 (15)	0.26484 (9)	0.09974 (7)	0.0320 (3)	
C1	0.43555 (19)	0.22301 (11)	0.27747 (10)	0.0348 (4)	
C2	0.33193 (19)	0.31439 (11)	0.16711 (9)	0.0350 (4)	
C3	0.28998 (18)	0.25375 (11)	0.24091 (9)	0.0336 (4)	
H3	0.2349	0.1963	0.2210	0.040*	
C4	0.2006 (2)	0.30886 (12)	0.30184 (10)	0.0409 (4)	
H4A	0.1979	0.2725	0.3541	0.049*	
H4B	0.2479	0.3709	0.3139	0.049*	
C5	0.0492 (2)	0.32639 (13)	0.27024 (11)	0.0485 (5)	
H5	0.0365	0.3419	0.2135	0.058*	
C6	−0.0667 (3)	0.32235 (16)	0.31310 (14)	0.0658 (6)	
H6A	−0.0595	0.3071	0.3701	0.079*	
H6B	−0.1585	0.3347	0.2874	0.079*	
C7	0.43855 (17)	0.10939 (11)	0.16252 (9)	0.0294 (3)	
C8	0.37894 (16)	0.16300 (10)	0.09694 (9)	0.0285 (3)	
C9	0.32362 (17)	0.11561 (12)	0.02736 (9)	0.0332 (4)	
H9	0.2852	0.1515	−0.0180	0.040*	
C10	0.32381 (18)	0.01744 (12)	0.02331 (10)	0.0371 (4)	
H10	0.2853	−0.0140	−0.0245	0.045*	
C11	0.38017 (18)	−0.03554 (12)	0.08892 (10)	0.0361 (4)	
H11	0.3788	−0.1033	0.0866	0.043*	
C12	0.43813 (17)	0.01011 (11)	0.15748 (10)	0.0331 (4)	
H12	0.4784	−0.0265	0.2019	0.040*	
C13	0.65246 (19)	0.12564 (13)	0.25940 (10)	0.0407 (4)	
H13A	0.6512	0.0580	0.2772	0.049*	
H13B	0.6853	0.1648	0.3073	0.049*	
C14	0.75779 (18)	0.13696 (12)	0.19117 (10)	0.0382 (4)	
C15	0.7462 (2)	0.21035 (14)	0.13427 (12)	0.0483 (5)	
H15	0.6699	0.2554	0.1376	0.058*	
C16	0.8450 (2)	0.21874 (16)	0.07231 (13)	0.0603 (6)	
H16	0.8353	0.2689	0.0331	0.072*	
C17	0.9570 (2)	0.15453 (16)	0.06759 (14)	0.0606 (6)	
H17	1.0243	0.1602	0.0250	0.073*	
C18	0.9714 (2)	0.08237 (15)	0.12436 (14)	0.0564 (5)	
H18	1.0498	0.0389	0.1218	0.068*	
C19	0.8714 (2)	0.07300 (13)	0.18536 (12)	0.0459 (5)	
H19	0.8808	0.0220	0.2238	0.055*	
C20	0.4289 (2)	0.32016 (12)	0.02996 (10)	0.0380 (4)	
H20A	0.4780	0.2757	−0.0073	0.046*	
H20B	0.5023	0.3659	0.0516	0.046*	
C21	0.3168 (2)	0.37497 (12)	−0.02019 (9)	0.0385 (4)	
C22	0.3657 (3)	0.43780 (13)	−0.07970 (11)	0.0553 (5)	
H22	0.4664	0.4466	−0.0858	0.066*	
C23	0.2682 (4)	0.48731 (17)	−0.12977 (13)	0.0764 (8)	
H23	0.3021	0.5301	−0.1702	0.092*	
C24	0.1224 (4)	0.47491 (18)	−0.12138 (15)	0.0839 (9)	
H24	0.0558	0.5084	−0.1566	0.101*	
C25	0.0723 (3)	0.41385 (16)	−0.06193 (15)	0.0704 (7)	
H25	−0.0286	0.4058	−0.0557	0.084*	
C26	0.1706 (2)	0.36411 (13)	−0.01120 (11)	0.0487 (5)	
H26	0.1363	0.3224	0.0299	0.058*	

### Atomic displacement parameters (Å^2^)

**Table:**

	*U*^11^	*U*^22^	*U*^33^	*U*^12^	*U*^13^	*U*^23^
O1	0.0684 (9)	0.0398 (7)	0.0320 (6)	−0.0053 (6)	−0.0109 (6)	−0.0058 (5)
O2	0.0876 (10)	0.0251 (7)	0.0365 (7)	−0.0007 (6)	0.0008 (7)	−0.0003 (5)
N1	0.0420 (8)	0.0286 (7)	0.0280 (7)	−0.0001 (6)	−0.0064 (6)	0.0014 (6)
N2	0.0451 (8)	0.0256 (7)	0.0254 (7)	0.0015 (6)	0.0000 (6)	0.0024 (5)
C1	0.0534 (11)	0.0253 (8)	0.0255 (8)	−0.0046 (8)	−0.0026 (7)	0.0035 (7)
C2	0.0494 (11)	0.0275 (9)	0.0277 (8)	0.0007 (8)	−0.0044 (7)	0.0003 (7)
C3	0.0496 (10)	0.0258 (8)	0.0251 (8)	0.0005 (7)	−0.0003 (7)	−0.0025 (6)
C4	0.0616 (12)	0.0328 (9)	0.0283 (9)	0.0048 (9)	0.0038 (8)	−0.0028 (7)
C5	0.0664 (14)	0.0411 (11)	0.0381 (10)	0.0129 (10)	0.0013 (9)	−0.0056 (8)
C6	0.0641 (15)	0.0742 (16)	0.0593 (13)	−0.0053 (12)	0.0039 (11)	−0.0084 (12)
C7	0.0322 (9)	0.0282 (8)	0.0277 (8)	−0.0006 (7)	0.0018 (7)	−0.0014 (6)
C8	0.0338 (9)	0.0250 (8)	0.0268 (8)	0.0005 (7)	0.0042 (7)	−0.0010 (6)
C9	0.0353 (9)	0.0374 (10)	0.0267 (8)	0.0040 (7)	−0.0012 (7)	−0.0031 (7)
C10	0.0361 (10)	0.0390 (10)	0.0363 (9)	−0.0002 (8)	0.0008 (7)	−0.0131 (8)
C11	0.0394 (10)	0.0270 (8)	0.0425 (10)	0.0000 (7)	0.0090 (8)	−0.0064 (7)
C12	0.0365 (9)	0.0287 (9)	0.0343 (9)	0.0029 (7)	0.0040 (7)	0.0029 (7)
C13	0.0466 (11)	0.0368 (10)	0.0379 (9)	0.0003 (8)	−0.0112 (8)	0.0053 (8)
C14	0.0378 (10)	0.0328 (9)	0.0430 (10)	−0.0051 (8)	−0.0135 (8)	0.0008 (8)
C15	0.0398 (11)	0.0435 (11)	0.0613 (12)	−0.0011 (9)	−0.0048 (9)	0.0144 (9)
C16	0.0472 (12)	0.0683 (15)	0.0654 (14)	−0.0086 (11)	0.0005 (10)	0.0253 (11)
C17	0.0401 (12)	0.0711 (15)	0.0707 (14)	−0.0050 (11)	0.0047 (10)	0.0036 (12)
C18	0.0400 (11)	0.0509 (13)	0.0779 (15)	0.0021 (9)	−0.0041 (11)	−0.0037 (11)
C19	0.0426 (11)	0.0368 (10)	0.0572 (12)	−0.0012 (9)	−0.0127 (9)	0.0023 (9)
C20	0.0502 (11)	0.0339 (9)	0.0302 (8)	−0.0051 (8)	0.0038 (8)	0.0057 (7)
C21	0.0631 (13)	0.0280 (9)	0.0243 (8)	0.0036 (8)	−0.0005 (8)	−0.0006 (7)
C22	0.0937 (16)	0.0383 (11)	0.0345 (10)	0.0081 (11)	0.0103 (10)	0.0069 (8)
C23	0.141 (3)	0.0484 (14)	0.0396 (12)	0.0187 (16)	0.0015 (14)	0.0142 (10)
C24	0.132 (3)	0.0580 (16)	0.0587 (15)	0.0313 (17)	−0.0366 (17)	0.0071 (12)
C25	0.0779 (16)	0.0554 (14)	0.0756 (16)	0.0177 (12)	−0.0285 (13)	−0.0023 (12)
C26	0.0633 (14)	0.0394 (11)	0.0425 (10)	0.0056 (10)	−0.0105 (9)	0.0016 (8)

### Geometric parameters (Å, °)

**Table:**

O1—C1	1.2271 (19)	C13—C14	1.509 (2)
O2—C2	1.2216 (19)	C13—H13A	0.9900
N1—C1	1.367 (2)	C13—H13B	0.9900
N1—C7	1.4321 (19)	C14—C15	1.383 (2)
N1—C13	1.475 (2)	C14—C19	1.388 (2)
N2—C2	1.3678 (19)	C15—C16	1.388 (3)
N2—C8	1.4273 (19)	C15—H15	0.9500
N2—C20	1.4710 (19)	C16—C17	1.377 (3)
C1—C3	1.516 (2)	C16—H16	0.9500
C2—C3	1.529 (2)	C17—C18	1.371 (3)
C3—C4	1.521 (2)	C17—H17	0.9500
C3—H3	1.0000	C18—C19	1.385 (3)
C4—C5	1.496 (3)	C18—H18	0.9500
C4—H4A	0.9900	C19—H19	0.9500
C4—H4B	0.9900	C20—C21	1.506 (2)
C5—C6	1.300 (3)	C20—H20A	0.9900
C5—H5	0.9500	C20—H20B	0.9900
C6—H6A	0.9500	C21—C26	1.376 (3)
C6—H6B	0.9500	C21—C22	1.393 (2)
C7—C12	1.393 (2)	C22—C23	1.380 (3)
C7—C8	1.400 (2)	C22—H22	0.9500
C8—C9	1.392 (2)	C23—C24	1.372 (4)
C9—C10	1.376 (2)	C23—H23	0.9500
C9—H9	0.9500	C24—C25	1.381 (4)
C10—C11	1.385 (2)	C24—H24	0.9500
C10—H10	0.9500	C25—C26	1.392 (3)
C11—C12	1.376 (2)	C25—H25	0.9500
C11—H11	0.9500	C26—H26	0.9500
C12—H12	0.9500		
			
C1—N1—C7	122.36 (14)	N1—C13—C14	112.15 (13)
C1—N1—C13	119.35 (13)	N1—C13—H13A	109.2
C7—N1—C13	118.29 (13)	C14—C13—H13A	109.2
C2—N2—C8	122.66 (13)	N1—C13—H13B	109.2
C2—N2—C20	117.67 (13)	C14—C13—H13B	109.2
C8—N2—C20	119.58 (12)	H13A—C13—H13B	107.9
O1—C1—N1	123.00 (16)	C15—C14—C19	118.41 (17)
O1—C1—C3	121.99 (15)	C15—C14—C13	122.04 (16)
N1—C1—C3	114.85 (13)	C19—C14—C13	119.55 (16)
O2—C2—N2	121.55 (15)	C14—C15—C16	120.56 (18)
O2—C2—C3	122.56 (14)	C14—C15—H15	119.7
N2—C2—C3	115.74 (13)	C16—C15—H15	119.7
C1—C3—C4	112.72 (13)	C17—C16—C15	120.13 (19)
C1—C3—C2	102.56 (13)	C17—C16—H16	119.9
C4—C3—C2	112.70 (13)	C15—C16—H16	119.9
C1—C3—H3	109.5	C18—C17—C16	120.0 (2)
C4—C3—H3	109.5	C18—C17—H17	120.0
C2—C3—H3	109.5	C16—C17—H17	120.0
C5—C4—C3	112.65 (14)	C17—C18—C19	119.90 (19)
C5—C4—H4A	109.1	C17—C18—H18	120.1
C3—C4—H4A	109.1	C19—C18—H18	120.1
C5—C4—H4B	109.1	C18—C19—C14	120.98 (18)
C3—C4—H4B	109.1	C18—C19—H19	119.5
H4A—C4—H4B	107.8	C14—C19—H19	119.5
C6—C5—C4	126.20 (19)	N2—C20—C21	116.17 (14)
C6—C5—H5	116.9	N2—C20—H20A	108.2
C4—C5—H5	116.9	C21—C20—H20A	108.2
C5—C6—H6A	120.0	N2—C20—H20B	108.2
C5—C6—H6B	120.0	C21—C20—H20B	108.2
H6A—C6—H6B	120.0	H20A—C20—H20B	107.4
C12—C7—C8	119.37 (14)	C26—C21—C22	119.19 (18)
C12—C7—N1	119.39 (14)	C26—C21—C20	123.27 (15)
C8—C7—N1	121.20 (14)	C22—C21—C20	117.52 (18)
C9—C8—C7	119.02 (14)	C23—C22—C21	120.2 (2)
C9—C8—N2	119.64 (14)	C23—C22—H22	119.9
C7—C8—N2	121.34 (13)	C21—C22—H22	119.9
C10—C9—C8	120.93 (15)	C24—C23—C22	120.2 (2)
C10—C9—H9	119.5	C24—C23—H23	119.9
C8—C9—H9	119.5	C22—C23—H23	119.9
C9—C10—C11	119.97 (15)	C23—C24—C25	120.2 (2)
C9—C10—H10	120.0	C23—C24—H24	119.9
C11—C10—H10	120.0	C25—C24—H24	119.9
C12—C11—C10	119.91 (15)	C24—C25—C26	119.6 (3)
C12—C11—H11	120.0	C24—C25—H25	120.2
C10—C11—H11	120.0	C26—C25—H25	120.2
C11—C12—C7	120.77 (15)	C21—C26—C25	120.5 (2)
C11—C12—H12	119.6	C21—C26—H26	119.8
C7—C12—H12	119.6	C25—C26—H26	119.8
			
C7—N1—C1—O1	178.80 (15)	C7—C8—C9—C10	1.6 (2)
C13—N1—C1—O1	−0.6 (2)	N2—C8—C9—C10	−179.23 (15)
C7—N1—C1—C3	−5.8 (2)	C8—C9—C10—C11	−0.3 (2)
C13—N1—C1—C3	174.81 (13)	C9—C10—C11—C12	−1.1 (2)
C8—N2—C2—O2	177.22 (16)	C10—C11—C12—C7	1.2 (2)
C20—N2—C2—O2	0.8 (2)	C8—C7—C12—C11	0.1 (2)
C8—N2—C2—C3	1.5 (2)	N1—C7—C12—C11	−177.57 (14)
C20—N2—C2—C3	−174.87 (14)	C1—N1—C13—C14	−124.15 (16)
O1—C1—C3—C4	−19.6 (2)	C7—N1—C13—C14	56.45 (19)
N1—C1—C3—C4	164.96 (14)	N1—C13—C14—C15	34.5 (2)
O1—C1—C3—C2	101.85 (17)	N1—C13—C14—C19	−146.39 (15)
N1—C1—C3—C2	−73.59 (16)	C19—C14—C15—C16	0.8 (3)
O2—C2—C3—C1	−99.46 (19)	C13—C14—C15—C16	179.95 (18)
N2—C2—C3—C1	76.21 (17)	C14—C15—C16—C17	−0.8 (3)
O2—C2—C3—C4	22.0 (2)	C15—C16—C17—C18	−0.3 (3)
N2—C2—C3—C4	−162.32 (14)	C16—C17—C18—C19	1.4 (3)
C1—C3—C4—C5	−171.39 (14)	C17—C18—C19—C14	−1.3 (3)
C2—C3—C4—C5	73.10 (19)	C15—C14—C19—C18	0.2 (3)
C3—C4—C5—C6	140.7 (2)	C13—C14—C19—C18	−178.88 (17)
C1—N1—C7—C12	−129.21 (16)	C2—N2—C20—C21	−71.74 (19)
C13—N1—C7—C12	50.2 (2)	C8—N2—C20—C21	111.77 (17)
C1—N1—C7—C8	53.2 (2)	N2—C20—C21—C26	−9.8 (2)
C13—N1—C7—C8	−127.46 (16)	N2—C20—C21—C22	171.75 (15)
C12—C7—C8—C9	−1.5 (2)	C26—C21—C22—C23	−1.0 (3)
N1—C7—C8—C9	176.16 (14)	C20—C21—C22—C23	177.51 (17)
C12—C7—C8—N2	179.35 (14)	C21—C22—C23—C24	−0.1 (3)
N1—C7—C8—N2	−3.0 (2)	C22—C23—C24—C25	1.0 (4)
C2—N2—C8—C9	134.46 (16)	C23—C24—C25—C26	−0.8 (4)
C20—N2—C8—C9	−49.2 (2)	C22—C21—C26—C25	1.2 (3)
C2—N2—C8—C7	−46.4 (2)	C20—C21—C26—C25	−177.19 (17)
C20—N2—C8—C7	129.94 (16)	C24—C25—C26—C21	−0.4 (3)
